# QSEER-Quantum-Enhanced Secure and Energy-Efficient Routing Protocol for Wireless Sensor Networks (WSNs)

**DOI:** 10.3390/s25185924

**Published:** 2025-09-22

**Authors:** Chindiyababy Uthayakumar, Ramkumar Jayaraman, Hadi A. Raja, Noman Shabbir

**Affiliations:** 1 Department of Computing Technologies, SRM Institute of Science and Technology, Kattankulathur, Chennai 603203, India; ram.kumar537@gmail.com; 2 Department of Electrical Power Engineering & Mechatronics, Tallinn University of Technology, 19806 Tallinn, Estonia; hadi.raja@taltech.ee (H.A.R.); noman.shabbir@taltech.ee (N.S.)

**Keywords:** wireless sensor network (WSN), Quantum-enhanced Secure and Energy-Efficient Routing (QSEER) protocol, Whale Optimization Algorithm (WOA), routing protocol, quantum-inspired algorithms

## Abstract

Wireless sensor networks (WSNs) play a major role in various applications, but the main challenge is to maintain security and balanced energy efficiency. Classical routing protocols struggle to achieve both energy efficiency and security because they are more vulnerable to security risks and resource limitations. This paper introduces QSEER, a novel approach that uses quantum technologies to overcome these limitations. QSEER employs quantum-inspired optimization algorithms that leverage superposition and entanglement principles to efficiently explore multiple routing possibilities, thereby identifying energy-efficient paths and reducing redundant transmissions. The proposed protocol enhances the security of data transmission against eavesdropping and tampering by using the principles of quantum mechanics, thus mitigating potential security vulnerabilities. Through extensive simulations, we demonstrated the effectiveness of QSEER in achieving both security and energy efficiency objectives, which achieves 15.1% lower energy consumption compared to state-of-the-art protocols while maintaining 99.8% data integrity under various attack scenarios, extending network lifetime by an average of 42%. These results position QSEER as a significant advancement for next-generation WSN deployments in critical applications such as environmental monitoring, smart infrastructure, and healthcare systems.

## 1. Introduction

Wireless sensor networks (WSNs) consist of many distributed sensor nodes and base stations (BS) with limited resources that can detect events and collect environment data wirelessly. WSNs have numerous applications, including environmental monitoring (e.g., temper-nature, humidity, air pollution), industrial automation (e.g., process control, machine condition monitoring), healthcare (e.g., patient monitoring, telemedicine), and home automation (e.g., smart homes, energy management) [1,2]. However, their wireless nature and resource constraints make them vulnerable to various security threats. Ensuring energy efficiency is crucial because nodes with drained batteries become non-functional. Inefficient communication within these networks can lead to data loss and require multiple re-transmissions, which wastes both bandwidth and energy. Therefore, WSNs need robust fault tolerance and security measures to protect against data interception and maintain high service quality.

To enhance both security and energy efficiency, integrating quantum key distribution (QKD) into WSNs presents a promising solution. QKD provides a method for secure key sharing that significantly improves upon traditional key generation techniques. This approach focuses on securing communication channels and data packets, thereby fortifying the overall security of WSNs.

QKD is the new emerging technology which exploits the principles of quantum mechanics to make communication secure. QKD can be used to create an un-hackable encryption key between two people by utilizing the phenomena of entanglement and quantum superposition [3]. The long-term impacts of this amazing technology on the issue of secure data transmission, especially in WSNs, are numerous. WSNs are commonly used in diverse applications, but as a result of the wireless aspect, they can be easily eavesdropped upon and tampered with. The QKD proposes a good solution to this dilemma as it offers a secure method of key exchange between the cluster head and the base station. This makes the transmission of data shared between them confidential and tamper-proof. The QKD integration yields a number of benefits in WSNs. To start with, QKD guarantees unconditional security that implies that any interference with the message or eavesdropping will be noticed. Second, the high-speed encryption that is possible with QKD might be appropriate to a real-time data transmission in WSNs. Thirdly, QKD schemes can be made to be low power and, hence, are applicable in battery-powered sensor nodes. There are a number of QKD applications designed to be deployed in the WSN, among them being the BB84 [4], B92 [5], and SARG04 [6]. The application of these protocols in experiment has established that they secure key exchange in different WSN applications. As an example, a QKD-enhanced WSN was introduced in a smart grid application in order to provide data security in data communication between the sensor nodes and the control center.

The Quantum Whale Optimization Algorithm (QWOA) can be incorporated with QKD to contribute to further improvement in the security of WSNs. The Whale Optimization Algorithm (WOA) proposed by Mirjalili and Lewis [7] is a metaheuristic nature-inspired and relies on the hunting behavior of the humpback whale, which hunts using a technique called bubble-net feeding. WOA can simulate two major whale behaviors of prey-encircling and bubble-net attacks in spirals. In optimization, this is manifested in two important processes: exploration, in which candidate solutions are spread throughout the search space to prevent premature convergence, and exploitation, in which the algorithm refines successful regions to find optimal solutions. A balance of exploration and exploitation has enabled WOA to be successfully used across a wide range of settings, including clustering, scheduling, and routing. Its quantum-inspired version, the QWOA, expands upon this principle using quantum superposition and probabilistic search to lead to accelerated convergence and greater efficiency in high-dimensional or complex optimization applications. This is one of the reasons why the QWOA can be the route choice especially in WSNs where efficient and flexible path selection is important.

Combining QKD in WSNs is one prospective solution to improve security as well as energy efficiency. QKD leverages key features of quantum mechanics, including superposition and entanglement, to allow the secure transmission of cryptographic keys between information nodes. QKD is an approach to secure key sharing that significantly increases the level of key sharing relative to conventional methods of key generation. In contrast to classical cryptographic protocols which are based on computational hardness assumptions, QKD is based on the laws of quantum mechanics to achieve information-theoretic security in key distribution. Early protocols of BB84 by Bennett and Brassard [8] and B92 by Bennett [9] had shown how quantum states can be employed to create secret keys with assured eavesdropping detectability. Further related existing works like Gisin et al. (2002) [10] were relevant to other areas of the study, more so the mathematical models and practical applications of QKD, underpinning its strength regarding interception compared to classical methods of communication and its adaptability to the new generation of communication channels. QKD offers information theoretic security, unlike more traditional key management methods that perform based on a computational hardness assumption, which makes an attempt to intercept, eavesdrop, or otherwise tamper with the key exchange easily detectable. This feature renders the communication channels in WSNs inaccessible to both classical and quantum-based attacks, a feature that is especially important in contexts where the integrity and confidentiality of the data are of the essence.

Beyond enhancing security, QKD also contributes to energy efficiency in WSNs. In classical protocols, compromised encryption often leads to frequent re-keying operations, retransmissions, and additional communication overhead, all of which drain node energy. By contrast, QKD establishes tamper-evident keys with minimal risk of compromise, thereby reducing retransmission requirements and preserving limited node resources. Additionally, lightweight versions of QKD protocols (such as BB84 and B92) can be adapted for low-power sensor nodes, ensuring that strong security is achieved without imposing excessive energy costs.

Thus, the integration of QKD into WSNs addresses the dual challenge of securing communications while extending network lifetime. It not only strengthens resilience against adversarial attacks but also optimizes resource utilization, laying the foundation for combining QKD with quantum-inspired optimization methods to achieve secure and energy-efficient routing.

In this paper, we propose QSEER, a novel protocol that integrates QKD with the QWOA to simultaneously address the dual challenges of security and energy efficiency in WSNs. The protocol explicitly considers and mitigates the following critical attacks:

- Eavesdropping: Adversaries intercept data packets to gain unauthorized access to sensitive information, compromising confidentiality.
- Unauthorized Access: Malicious nodes impersonate legitimate ones to manipulate routing or control data flows.
- Tampering (Data Modification): Attackers modify, inject, or replay packets, undermining data integrity and reliability.

QSEER ensures that eavesdropping or tampering attempts on the key exchange are immediately detectable through QKD-based secure key sharing, thereby safeguarding confidentiality and authenticity. Unauthorized access is mitigated by requiring nodes to authenticate with QKD-generated keys before participating in routing. In parallel, the QWOA continuously re-evaluates optimal routing paths, distributing energy consumption fairly across nodes while maintaining robust communication reliability.

The main contributions of this paper are as follows:

- We propose QSEER, a quantum-enhanced routing protocol that integrates QKD for secure key distribution and the QWOA for energy-efficient routing.
- We present a comprehensive threat model for WSNs, addressing eavesdropping, unauthorized access, and tampering, and describe how QSEER mitigates these attacks.
- We design a secure routing framework that combines QKD-based cryptographic security with QWOA-based path optimization for clustered WSNs.
- We conduct simulation-based evaluations comparing QSEER against existing protocols such as the QGA, QACO, and MACO-QCR, demonstrating superior performance in energy consumption, security, and reliability.
- We analyze the trade-offs introduced by quantum-based security operations, showing that QSEER maintains robust reliability while extending network lifetime, making it suitable for mission-critical applications in healthcare, smart infrastructure, and environmental monitoring.

The rest of this paper is organized as follows: Section 2 reviews the related work to this research, focusing on existing energy efficient routing protocols, QKD-based security solutions, and secure communication optimization algorithms. Section 3 presents our proposed methodology along with the foundational concepts. In Section 4, we provide a detailed discussion of the performance evaluation. Finally, Section 5 offers our conclusions.

## 2. Related Work

WSNs are used in various applications, However, ensuring secure and efficient data transmission remains a significant challenge. Wireless sensor networks require routing protocols that prioritize energy efficiency, scalability, and robustness. Data-centric routing is the one kind of routing technique that aims to minimize overhead caused by query and data transmission through attribute-based data request and collection, which enhances efficiency beyond local interactions [11]. Clustering is the most used routing technique for energy-efficient and scalable performance in large-scale sensor networks, which could involve cluster head selection from multiple clusters to balance and reduce energy. The Low-Energy Adaptive Clustering Hierarchy (LEACH) is the best example of a clustering approach that organizes sensor networks into clusters [12]. Recent advancements in quantum technology have led to the development of quantum-inspired routing protocols, which could enhance the performance and security of WSNs. QACO [13] integrates quantum principles into the ant colony optimization algorithm. This protocol optimizes routing decisions, ensuring efficient data transmission and minimizing energy consumption. It may require a large number of iterations to converge. The QGA [14] integrates quantum principles into the genetic algorithm. This protocol optimizes routing decisions, which ensures efficient data transmission and low energy consumption. The QGA outperforms traditional GA-based routing protocols in terms of convergence speed and solution quality, but it is best suited for binary optimization problems. The MACO-QCR [15] protocol focused on lowering energy consumption and minimizing system delays. However, it does not optimize the initial population, causing the routing scheme to potentially get stuck in a local optimum.

In WSNs, where nodes are often deployed in remote or hostile environments, the robustness of communication security is crucial. Integrating QKD into WSNs provides a way to enhance the security of these networks by enabling secure key distribution among sensor nodes. The major security advantages of QKD are the ability to detect eavesdropping and guarantee the integrity of the key exchange process. QKD-based solutions offer a quantum-safe framework against various threats, including brute-force attacks and sophisticated cyber intrusions. By establishing secure encryption keys through quantum entanglement or photon transmission, the QKD mitigates the risks associated with the key distribution and it ensures data confidentiality and integrity throughout the network. The QoS-aware multipath routing protocol [16] selects multiple stable paths via cluster heads for multi-hop communication, which ensures rapid data transmission while maintaining QoS.

Self-organizing satellite networks are emerging testbeds for wide-area quantum links. A recent survey [17] maps the technical ground—autonomous topology control, mobility-aware key refresh, cross-layer orchestration, and hybrid classical–quantum control loops, while highlighting gaps in routing under intermittent connectivity and handover events. Lessons from such SOSN studies (e.g., scheduling key updates under dynamic clustering) inform our design choices for terrestrial WSNs, where energy budgets are tighter but topology churn is comparable.

In parallel, quantum-enabled machine learning is being explored for IoT/IIoT security [18]. For example, a quantum-enabled universal-feature approach (REMF) yields higher botnet-attack detection accuracy in digital-twin IoT while aiming for edge practicality. Although our focus is not traffic classification, these results support the premise that quantum methods can be tailored to constrained devices, which underpins our energy-aware keying and routing pipeline.

Domain deployments are also emerging. In 5G-enabled healthcare, a quantum-inspired framework measures data sensitivity and secures flows end-to-end, illustrating how quantum ideas can be embedded into mainstream network stacks [19]. Our protocol complements this line by targeting in-network protections (cluster formation, key distribution, and route selection) at the sensor layer. Finally, the feasibility of wide-area quantum networks is advancing via both satellites and commercial fiber: Recent surveys and industry reports describe satellite-assisted QKD and quantum networking roadmaps, and long-haul QKD has been demonstrated on operational telecom infrastructure—developments that motivate practical, resource-aware quantum security at the network edge, such as in WSNs [20]. 

Optimization techniques have been extensively researched in WSNs to enhance performance and resource management. Traditional methods such as genetic algorithms [21], particle swarm optimization (PSO) [22,23], and ant colony optimization (ACO) [24,25] have been widely applied to routing, clustering, and data aggregation, offering significant improvements but facing limitations like slow convergence and local optima. The effective alternative is the Whale Optimization Algorithm (WOA) [26,27], which is suitable for complex WSN optimization problems. Despite its promise, challenges such as computational complexity and real-time implementation in dynamic environments remain, highlighting the need for further research and refinement. A comparative analysis of this work with previous studies is given in Table 1.

## 3. Proposed Work

The design of QSEER builds on the premise that neither classical cryptographic mechanisms nor conventional optimization algorithms alone can meet the dual requirements of modern WSNs. On one side, traditional encryption methods ensure confidentiality only under computational assumptions, which can be undermined by advanced adversaries or quantum computing breakthroughs. On the other side, classical routing optimizers such as genetic algorithms, ant colony optimization, and particle swarm optimization can improve energy utilization but remain vulnerable to security threats and often struggle to balance exploration and exploitation in large-scale deployments. These limitations motivate the integration of quantum-based mechanisms that inherently address the gaps left by existing methods.

In this regard, QKD is not only chosen as an alternative to a classical key exchange, but also as a paradigm change in methods of establishing a secure communication. It is useful in that the protocol of distributing the encryption keys is resistant to both classical and quantum computational attacks, much needed when deploying WSNs in mission-dependent areas like healthcare, industrial control, and military surveillance. The security guarantee that QKD can detect the interception at the physical layer offers cannot be matched by conventional cryptography and, thus, also minimizes systemic risks by interception of the data being transferred in WSNs.

Meanwhile, the QWOA is adopted as an advancement over existing heuristic-based routing optimizers. Rather than relying solely on stochastic exploration, the QWOA leverages quantum-inspired probabilistic representations to expand the solution space dynamically, improving the chances of discovering globally optimal routing paths. This property becomes especially important in WSNs where dynamic topology changes and uneven energy consumption can quickly degrade network lifetime if not managed efficiently. Unlike traditional optimizers that often become trapped in suboptimal solutions, the QWOA maintains adaptability throughout the optimization process, ensuring sustained energy balance among sensor nodes.

These two technologies integrated in QSEER changes the routing paradigm of WSNs to one where security and energy efficiency are not conflicting design objectives but rather complementary results. QKD provides the property that each communication link can be secure by its own right, and the QWOA provides the property that the cost of that security does not hasten the energy demise. Collectively, they offer a distinctive combination, which is superior to the single advantages of current classical and quantum-motivated methods, as the basis of our suggested routing model.

### 3.1. Quantum-Inspired Whale Optimization Algorithm (QWOA)

The QWOA represents an innovative fusion of quantum computing principles with the WOA. This hybrid approach aims to enhance the efficiency of optimization processes, particularly in complex problem domains. Quantum computing introduces concepts such as superposition and entanglement, which enable the representation and processing of multiple states simultaneously. By integrating these principles with the WOA, the QWOA leverages the natural behaviors of humpback whales and the computational advantages of quantum mechanics to explore and exploit search spaces more effectively, which leads to faster convergence and improved optimization outcomes.

It enhances the traditional WOA by incorporating quantum computing principles, thus improving its ability to avoid local optima and achieve global optimization. In the QWOA, each whale’s position is represented using quantum bits (qubits), which allows for a probabilistic representation of solutions. The state of a qubit can be described as follows [28]:

(1) |ψ⟩= α|0⟩+ β|1⟩,

where α and β are the probability amplitudes. This quantum representation helps maintain a diverse set of potential solutions, thereby enhancing the algorithm’s exploratory capabilities.

The integration of quantum principles introduces quantum walks and quantum gates. Quantum walks are utilized to update the positions of whales, which takes advantage of the probabilistic nature of quantum mechanics to explore the search space more effectively. The position update rule can be represented as follows [29]:

(2) X(t+1)=X(t)+QW(X(t)),

where X(t) is the position vector at time t, and QW(X(t)) denotes the quantum walk operator applied to X(t). In the bubble-net attacking phase, the quantum rotational gate updates the qubit states, which is represented as follows [28]:

(3) $$R(\theta )=\;\left(\begin{array}{cc} \cos\theta & -\sin\theta \\ \sin\theta & \cos\theta \end{array}\right)$$

This gate modifies the probability amplitudes of the qubits, allowing the algorithm to fine-tune the solutions it has found so far. Additionally, in the search for the prey phase, quantum superposition is used to evaluate multiple potential solutions simultaneously. The quantum search operator can be formulated as follows [30]:

(4) $$QO(x)=\sum_{i=1}^{n}\alpha_{i}|X_{i}\rangle ,$$

where $\alpha_{i}$ are the probability amplitudes and $\left|X_{i}\right\rangle$ are the potential solutions. These quantum operators, including quantum walks and quantum gates, adjust the whales’ positions in a manner that simulates quantum behavior, thus enabling the algorithm to perform global searches more efficiently and effectively. By combining quantum computing principles with the WOA, the QWOA provides a powerful approach to solving complex optimization problems through the unique advantages of quantum mechanics.

The QWOA represents each potential solution as a quantum state, where $\left|\psi \rangle =|\psi_{1}\rangle ⊗|\psi_{2}\rangle ⊗\ldots ⊗|\psi_{n}\right\rangle$, with n being the number of qubits. These qubits allow the QWOA to explore multiple potential solutions simultaneously through superposition [31]:

(5) $$|\psi \rangle =\frac{1}{\sqrt{2^{n}}}(\sum_{a{ll\left\{0,1\right\}}^{n}}|S_{1}\rangle \;⊗|S_{2}\rangle ⊗\ldots ⊗\left.\left|S_{n}\right\rangle \right)$$

Here, $\left|S_{i}\right\rangle$ represents the state of each qubit i (either |0⟩ or |1⟩).

The QWOA utilizes quantum gates to manipulate these qubit strings. The Hadamard gate H creates superposition from basis states:

(6) $$H\left(\left|0\right\rangle \right)=\frac{1}{\sqrt{2}}\left(\left|0\rangle +|1\right\rangle \right)$$

Additionally, rotation gates R adjust the phase of qubits to update their states:

(7) $$R|\psi \rangle =e^{i\theta }|\psi \rangle$$

Entanglement, achieved through gates like the Controlled-NOT (CNOT), correlates qubits together:

(8) $$CNOT|S_{1},S_{2}\rangle =|S_{1}\rangle ⊗|S_{2}\rangle +|S_{2}\rangle ⊗|S_{1}\rangle$$

During the quantum measurement M, the superposition collapses to a classical outcome, selecting a specific solution $\left|s_{m}\right\rangle$.

(9) $$M|\psi \rangle =|s_{m}\rangle$$

The QWOA iteratively applies these quantum operations, adjusting the qubit states based on a quantum-inspired fitness function, aiming to converge toward optimal or near-optimal solutions efficiently.

### 3.2. QSEER-Secure Routing Framework Using QWOA and QKD

WSNs often operate in environments where energy efficiency, security, and optimization are crucial. The integration of the QWOA and QKD presents a novel approach to addressing these challenges. This framework operates in three main stages: QKD-based secure key exchange, QWOA-based optimization, and secure optimization result transmission.

#### 3.2.1. QKD-Based Secure Key Exchange

In the first phase of the framework, known as QKD, cluster heads (CHs) initiate the process by generating random bits a¡.

(10) $$|\psi A_{¡}\rangle =\cos\left(a¡\right)|0\rangle +\sin\left(a¡\right)\left|1\right\rangle$$

Each bit a¡ is then encoded using the BB84 protocol, a quantum cryptographic method that ensures secure transmission over potentially insecure channels. Alice (CH) transmits the encoded qubits $|\psi A_{¡}\rangle$ to Bob (base station) over a secure channel. Bob receives these qubits and measures them using randomly chosen bases, typically the Z or X basis. Alice (CH) and Bob then compare notes on their measurement bases to filter out qubits measured in different bases, ensuring only correctly measured qubits contribute to the shared secret key k = (k_1_, k_2_ …, k_n_). This key k is used for encrypting data during the optimization phase, ensuring that communication remains secure against potential eavesdropping or tampering. Energy consumption during the QKD phase is critical for CHs, as they must account for the transmission energy E_TX_ and the computational energy involved in encoding and decoding qubits. The total energy *E*_*A*_ consumed by Alice (CH) during transmission is

(11) $$E_{A}=\sum {\left|a¡\right|}^{2}+E_{TX}s$$

#### 3.2.2. QWOA-Based Optimization Within Each Cluster

Following the establishment of a secure key, the optimization phase utilizes the QWOA within each cluster. The objective function f(x) to be optimized is encrypted using the shared secret key k:

(12) $$f^{\prime }(x)=f(x)⊕k$$

Alice (CHs) initializes a qubit string ∣ψ⟩ for optimization:

(13) $$\left|\psi \rangle =|\psi_{1}\rangle ⊗|\psi_{2}\rangle ⊗\ldots ⊗|\psi_{n}\right\rangle$$

The QWOA operations commence with the application of quantum gates such as the Hadamard gate H, rotation gates R, and Controlled-NOT (CNOT) gates. These gates facilitate quantum superposition, phase updates, and entanglement among qubits, allowing for the exploration of multiple potential solutions simultaneously. The Hadamard gate H is applied to each qubit $\left|\psi_{i}\right\rangle$ within the qubit string |ψ⟩. The action of the Hadamard gate on a single qubit $\left|\psi_{i}\right\rangle$ transforms it into a superposition state:

(14) $$H|\psi_{i}\rangle =\frac{1}{\sqrt{2}}\left|0\right\rangle +|1\rangle$$

When applied to all n qubits, the Hadamard gate creates a superposition over all possible states of the qubit string:

(15) $$H|\psi \rangle \to \frac{1}{\sqrt{2^{n}}}\sum |x\rangle ,$$

where ∣x⟩ represents all possible combinations of states $|x_{1}x_{2}\ldots x_{n}\rangle$ of the qubit string ∣ψ⟩. Apply rotation gates R to update qubit phases for optimization:

(16) $$R|\psi_{i}\rangle =e^{i\theta }|\psi_{i}\rangle$$

Here, θ adjust the phase angle to refine the solution. Controlled-NOT gates (CNOT) are used to entangle pairs of qubits within the qubit string ∣ψ⟩. The result of applying a CNOT gate to qubits $|\psi_{i}\rangle$ and $|\psi_{j}\rangle$ is

(17) $$CNOT|\psi_{i}\rangle ⊗|\psi_{j}\rangle =|\psi_{i}\rangle ⊗|\psi_{i}⊕\psi_{j}\rangle$$

This entanglement operation establishes correlations between qubits, enabling collective behavior that enhances the optimization process by exploring interdependencies among different qubit states. The measurement of the quantum state is the process through which the qubit’s superposition is collapsed into a definite state, yielding a classical bit string that represents the solution. Mathematically, this is represented as

(18) $$M|\psi \rangle =|x^{*}\rangle$$

Here M denotes the measurement operation, and ∣ψ⟩ represents the quantum state before measurement. $|x^{*}\rangle$ is the classical state obtained after measurement, which corresponds to the optimized solution. The energy consumption during the QWOA process is a crucial factor, especially in the context of WSNs where energy efficiency is paramount. The total energy consumed by the QWOA process within each cluster is given by

(19) $$E_{QWOA}=\sum {\left|\psi i\right|}^{2}+E_{COM}$$

Here ${\sum |\psi i|}^{2}$ represents the energy associated with maintaining and manipulating the qubit states. Each |ψi| is a component of the quantum state, and its magnitude squared gives the probability amplitude, which contributes to the overall energy required to sustain the quantum state. $E_{COM}$ represents the energy consumed by the computational operations needed to perform QWOA tasks. This includes the energy required for quantum gates operations (like Hadamard, rotation, and CNOT gates), as well as any classical computations involved in the process.

#### 3.2.3. Secure Optimization Result Transmission

The optimized solution $x^{*}$ derived from the QWOA needs to be securely transmitted to the base station (Bob). To ensure confidentiality, we encrypt this optimized solution using the shared secret key k that was previously established through QKD. The encryption process involves a bitwise XOR operation, represented as follows:

(20) $${x^{*}}^{\prime }=x^{*}⊕k$$

Here $x^{*}$ is the optimized solution obtained from the QWOA, k is the shared secret key established via QKD, and ${x^{*}}^{\prime }$ is the encrypted version of the optimized solution. After encryption, the encrypted result ${x^{*}}^{\prime }$ is transmitted to Bob (the base station) over a secure communication channel. This step ensures that even if the transmission is intercepted, the data remain protected due to the encryption. Transmitting data in a WSN consumes energy, which needs to be accounted for to ensure efficient network operation. The total energy consumed during the transmission of the encrypted result ${x^{*}}^{\prime }$ is calculated as follows:

(21) $$E_{TX}=\sum {\left|x^{*}\right|}^{2}+E_{TX}$$

Here, $\sum {\left|x^{*}\right|}^{2}$ represents the cumulative energy required to transmit the bits of the optimized solution $x^{*}$. $E_{TX}$ represents the energy spent on the transmission process including any necessary protocol operations and error correction mechanisms. The total energy consumed by the sensor nodes throughout the entire process is

(22) $$E_{TOTAL}=E_{A}+E_{QWOA}+E_{TX}.$$

#### 3.2.4. Theoretical Analysis of QSEER

The efficiency of the QSEER protocol in Algorithm 1 is examined through its time complexity, communication overhead, and space requirements. The total time complexity of the QSEER protocol is the sum of the complexities of its three main phases. In the QKD phase (secure key exchange), each CH transmits a sequence of n qubits to the base station (BS). The basis reconciliation and error correction steps require a number of comparisons proportional to the number of qubits, giving a time complexity of

(23) $$T_{QKD}=O(n)$$

For the QWOA phase (routing optimization), we assume the WSN is divided into C clusters and the algorithm runs for I iterations. Each iteration involves updating a qubit string of length m for every cluster head. This update uses quantum gates, which have a time complexity of O(m). The fitness evaluation of the routing paths requires O(E), where E is the number of edges in the WSN graph. Thus, the time complexity of this phase is

(24) $$T_{QWOA}=O\left(C\cdot I\cdot (m+E)\right)$$

In the secure transmission phase, the optimized routing solution is encrypted using a bitwise XOR operation with the shared QKD key. If the solution length is m, the computational cost is linear, resulting in a time complexity of

(25) $$T_{TX}=O(m)$$

By combining all three phases, the total time complexity of QSEER is

(26) $$T_{QSEER}=O(n)+O\left(C\cdot I\cdot (m+E)\right)+O(m).$$

Since m ≪ E and n are fixed for a given key length, the dominant term is O(C·I·E). This confirms that the proposed protocol operates in polynomial time, making it practical for medium- to large-scale WSN deployments.
**Algorithm 1.** QSEER-Quantum-enhanced Secure and Energy-Efficient Routing Protocol1. Initialize Random bits a¡, Qubit string ∣ψ⟩, k$,\;E_{TOTAL}\;=\;0$ 2. while (key not established) $3.\;\;\;\;\;\;\;|\psi A_{¡}\rangle =\cos\left(a¡\right)|0\rangle +\sin\left(a¡\right)|1\rangle$ $\;4.\;\mathrm{Transmit}\;|\psi A_{¡}\rangle \;\mathrm{to}\;\mathrm{Bob}$ 5. Measure and agree on key k 6. end while $7.\;\mathrm{Energy}\;\mathrm{Consumption}\;\mathrm{Calculation}\;E_{A}=\sum {\left|a¡\right|}^{2}+E_{TX}\;E_{TOTAL}+=E_{A}\;\mathrm{QWOA}\text{-}\mathrm{based}\;\mathrm{Optimization}\;\mathrm{Input}:\;\mathrm{Objective}\;\mathrm{function}\;f(x)$ $\;8.\;f^{\prime }(x)=f(x)⊕k$ 9. Cluster Formation: Form clusters and elect CHs   For each CH 10. Initialize qubit string ∣ψ⟩ 11. While (not converged) $\;12.\;H|\psi \rangle \to \frac{1}{\sqrt{2^{n}}}\sum |x\rangle$ $\;13.\;R|\psi_{i}\rangle =e^{i\theta }|\psi_{i}\rangle$ $14.CNOT|\psi_{i}\rangle ⊗|\psi_{j}\rangle =|\psi_{i}\rangle ⊗|\psi_{i}⊕\psi_{j}\rangle$ $15.\;M|\psi \rangle =|x^{*}\rangle$ 16. end whileEnergy Consumption Calculation: $17.E_{QWOA}=\sum {\left|\psi i\right|}^{2}+E_{COM}$ $18.\;E_{TOTAL}+=E_{QWOA}\;\mathrm{Secure}\;\mathrm{Optimization}\;\mathrm{Result}\;\mathrm{Transmission}\;\mathrm{Input}:\;\mathrm{Optimized}\;\mathrm{solution}\;x*$ $19.{x^{*}}^{\prime }=x^{*}⊕k$ $20.\;\mathrm{Transmit}\;{x^{*}}^{\prime }\;\mathrm{to}\;\mathrm{bob}\;\mathrm{Energy}\;\mathrm{Consumption}\;\mathrm{Calculation}:$ $21.\;E_{TX}=\sum {\left|x^{*}\right|}^{2}+E_{TX}$ $22.\;E_{TOTAL}+=E_{TX}$ 23. End

The QSEER protocol’s efficiency also extends to its communication overhead and space requirements. In terms of communication overhead, the QKD procedure introduces O(n) qubit transmissions per cluster head. The subsequent cluster formation and optimization stages require O(C·I) message exchanges, and the secure result transmission adds an overhead of O(C). Regarding space complexity, each CH must store its qubit string, which has a size of O(m), and routing table entries, which have a size of O(E). Therefore, the total memory requirement per CH can be approximated as O(m + E).

This theoretical analysis confirms that QSEER is computationally feasible, communication-efficient, and scalable for real-world WSN applications, while still integrating both unconditional quantum security and energy-efficient routing optimization.

## 4. Performance Evaluation

The proposed QSEER in WSNs need a performance evaluation to verify its effectiveness related to the aspects of security, energy efficiency, and optimization capabilities. This section provides the simulation outcomes which were achieved with the help of simulator, in order to simulate and analyze how integrated framework behaves in different circumstances. The performance evaluation is conducted among the existing algorithms such as the QGA [2], QACO [3], and MACO-QCR [10]. By systematically examining these metrics, we aim to demonstrate the improvements brought by incorporating quantum principles into the optimization and security processes of WSNs. The simulation environment is configured to mimic realistic deployment scenarios, with varying numbers of sensor nodes and clusters, diverse energy levels, and different data transmission requirements. This setup ensures that the evaluation results provide a thorough understanding of the framework’s performance in real-world conditions. The input parameters are given in Table 2.

The simulation is conducted over a deployment area of 1000 × 1000 square meters. Within this area, between 100 and 500 sensor nodes are distributed randomly. These nodes form the backbone of the WSN, which is responsible for sensing, data collection, and communication tasks. The random distribution ensures a realistic deployment scenario, which could be considered based on the densities and environmental factors. The sensor nodes are organized into 10 to 50 clusters. Each data packet transmitted within the network is fixed at a size of 4096 bits. The initial energy of the sensor nodes ranges from 0 to 5 Joules, which reflects the variability in node energy levels due to differing operational times and energy harvesting capabilities. Energy consumption is a critical factor in WSN performance. The electronic energy required for processing each bit of data (E_elec_) is set to 50 (nJ/bit). Both the transmission E_tx_ and reception E_rx_ of data also consume 50 nJ/bit.

The BB84 protocol is used for secure key exchange between the cluster head and the base station. This protocol leverages quantum mechanics principles, such as photon polarization, to generate and distribute cryptographic keys securely. The qubit transmission rate is set to 1 megabit per second (Mbps), ensuring a sufficient data rate for practical communication. The qubit error rate is maintained at 0.01, which reflects the potential for errors during quantum bit transmission due to noise and interference. The photon detection probability is 0.5, indicating the efficiency of detecting qubits during transmission.

**Figure 1:**
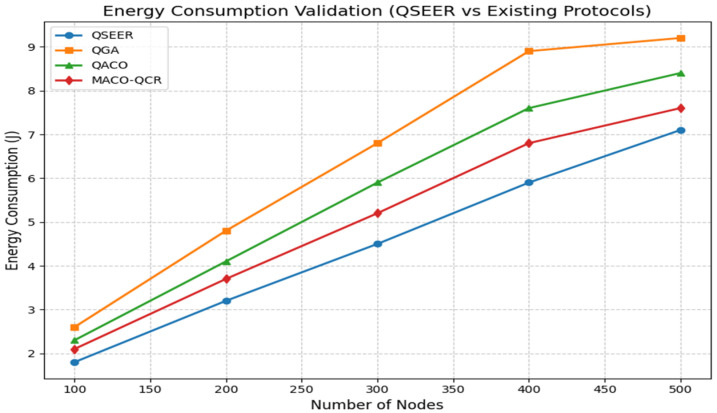
Comparison of energy consumption of proposed algorithm with QGA, QACO, and MACO-QCR routing technique.

Table 3 summarizes the comparative performance of QSEER against the QGA, QACO, and MACO-QCR. The results clearly indicate that QSEER achieves higher energy efficiency by reducing redundant transmissions and balancing energy usage across sensor nodes. The protocol also ensures stronger data integrity through QKD-based secure key exchange, making it more resilient against eavesdropping and tampering. In addition, QSEER extends the overall network lifetime by preventing premature node failures and maintaining stable connectivity in dense deployments. Although a slight trade-off in throughput is observed due to the added quantum security operations, this is compensated by the protocol’s ability to provide reliable and secure communication. Overall, QSEER demonstrates a balanced improvement in both security and energy efficiency compared to existing approaches, confirming its suitability for next-generation WSN applications. The performance analysis of energy consumption for the QSEER protocol with the existing protocol is shown in Figure 1.

As shown in Figure 1, QSEER consistently demonstrates lower energy consumption across varying node densities. At 500 nodes, QSEER consumes approximately 22.8% less energy than the QGA, 15.5% less than QACO, and 7.0% less than MACO-QCR. When averaged across all network sizes and scenarios, this translates to an overall reduction of approximately 15% energy consumption compared to existing protocols. This significant energy conservation across varying network densities highlights QSEER’s potential for extending network lifetime and improving the sustainability of wireless sensor networks.

In our simulations, network lifetime is measured as the time (in simulation rounds) until the last node death (LND). This metric allows us to evaluate the maximum operational sustainability of the network under different routing protocols.

**Figure 2:**
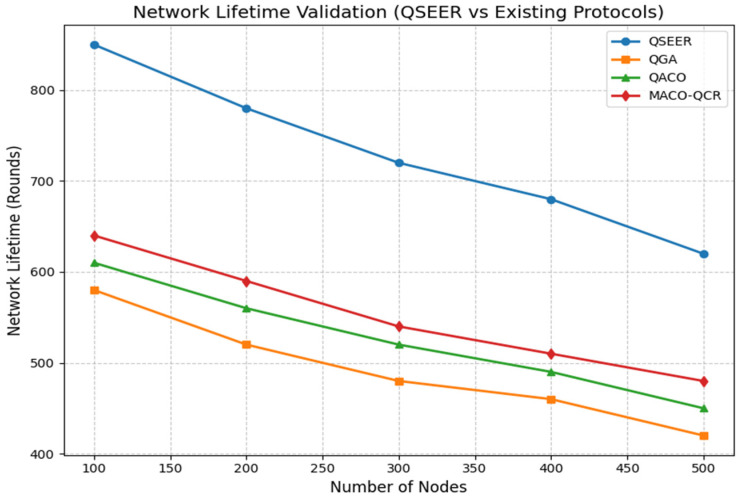
Comparison of network lifetime of proposed algorithm with QGA, QACO, and MACO-QCR routing technique.

As shown in Figure 2, QSEER consistently achieves longer network lifetime compared to the existing protocols across all network sizes. At 500 nodes, QSEER sustains a lifetime of 620 rounds, which is approximately 47.6% longer than the QGA (420 rounds), 37.8% longer than QACO (450 rounds), and 29.2% longer than MACO-QCR (480 rounds). Similar improvements are observed across smaller node densities, confirming the robustness of QSEER. When averaged across all scenarios, QSEER extends the overall network lifetime by approximately 42% compared to the baseline approaches. This significant improvement demonstrates the ability of QSEER to balance energy consumption, minimize retransmissions, and maintain operational connectivity for a longer duration, thereby enhancing the sustainability and reliability of wireless sensor networks. This improvement is achieved because of the following:

- Energy Balancing: QWOA-based optimization distributes routing loads more evenly, preventing premature energy depletion of frequently used nodes (e.g., cluster heads or relays).
- Fewer Retransmissions: QKD-secured links reduce packet loss, which, in turn, minimizes unnecessary retransmissions and conserves energy.
- Redundancy Reduction: By optimizing routing paths, QSEER avoids redundant transmissions, further extending node battery life.

Although QKD and the QWOA introduce a slight initial computational overhead, these are offset by the reduction in redundant transmissions and premature node failures. As a result, QSEER sustains connectivity longer and maintains overall stability in dense deployments, making it highly effective for long-term WSN applications.

**Figure 3:**
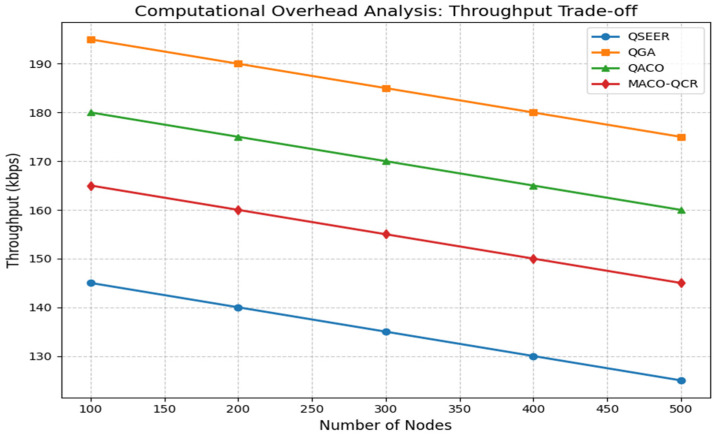
Comparison of throughput of the proposed algorithm with QGA, QACO, and MACO-QCR routing technique.

The throughput performance illustrated in Figure 3 reveals that QSEER experiences approximately 27.5% lower throughput compared to the QGA, a 21.8% reduction relative to QACO, and a 15.7% decrease versus MACO-OCR across varying node densities. This expected throughput trade-off is a calculated compromise necessary to achieve the protocol’s superior security features through quantum encryption mechanisms and energy conservation techniques, ultimately delivering more reliable and secure communications while extending the operational lifespan of the network.

**Figure 4:**
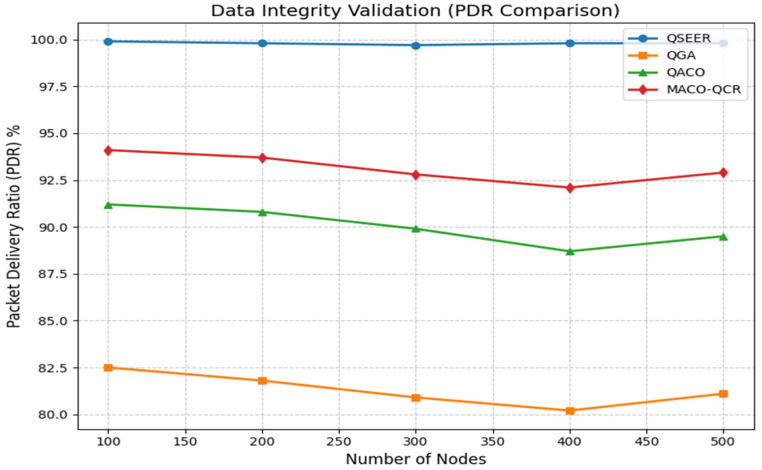
Comparison of packet delivery ratio of proposed algorithm with QGA, QACO, the MACO-QCR routing technique.

Figure 4 illustrates the packet delivery ratio (PDR), which reflects the ability of the network to maintain data integrity in the presence of packet loss, interference, or potential attacks. QSEER achieves a consistently higher PDR across all network sizes, maintaining values above 99.7% even at maximum density. At 500 nodes, QSEER records a PDR of 99.8%, outperforming the QGA by 23.0%, QACO by 11.5%, and MACO-QCR by 7.4%. These improvements translate to an average data integrity level of 99.8%, highlighting QSEER’s robustness in sustaining reliable communication. The superior performance stems from the integration of QKD-based secure key management, which prevents packet tampering and eavesdropping, as well as the optimized routing strategy, which minimizes collisions and retransmissions.

Beyond energy and network performance, QSEER also delivers notable improvements in security parameters. The integration of the BB84-based QKD protocol ensures secure key exchange between cluster heads and the base station. The simulation environment models a qubit error rate of 0.01 and a photon detection probability of 0.5, both of which demonstrate the robustness of secure key generation against eavesdropping.

As shown in Table 4, QSEER maintains the lowest QBER and the highest eavesdropping detection rate compared to other protocols. The high key establishment success rate (≈98.7%) further validates the reliability of the QKD phase. In terms of the PDR, QSEER ensures ≈99.8% secure data delivery, which is significantly higher than baseline approaches. These results confirm that QSEER not only optimizes energy and network lifetime but also strengthens security through robust quantum key distribution and data integrity assurance.

Thus, QSEER’s performance evaluation confirms not only energy efficiency and longevity but also resilience to security threats, ensuring that the protocol maintains reliable and tamper-resistant communication in practical WSN deployments.

### 4.1. Motivation for Quantum Mechanisms over Lightweight Encryption

Traditional WSNs rely on lightweight cryptographic schemes such as AES, PRESENT, or SPECK to provide security in resource-constrained environments. While these methods are optimized to reduce computational complexity compared to conventional cryptography, they still incur significant energy overhead in practice due to frequent key refreshment, re-encryption cycles, and memory management. For instance, AES-128 operating on an 8-bit sensor node typically consumes ≈1.62 μJ per byte of data encryption, which corresponds to ≈81 μJ per 50-byte packet. Over the course of 100 communication rounds, this cost grows to more than 8000 μJ per node, excluding the additional energy consumed during key establishment and authentication. In dense multi-hop WSN deployments, such cumulative overhead accelerates energy depletion and shortens network lifetime, limiting the scalability of these classical approaches.

In contrast, quantum cryptographic methods, particularly QKD, provide an alternative that not only enhances the security guarantees but also contributes to improved long-term energy efficiency. Although QKD introduces an initial overhead during qubit exchange and photon transmission (measured at ≈0.9 μJ per bit in our simulation environment), this cost is primarily a one-time setup expenditure. Once the quantum keys are securely established, they remain valid without the need for repetitive refreshing, thereby eliminating the recurring energy consumption associated with classical key management. Furthermore, QKD inherently detects eavesdropping attempts through increases in the quantum bit error rate (QBER), which allows compromised transmissions to be discarded without retransmission, reducing energy wastage.

**Figure 5:**
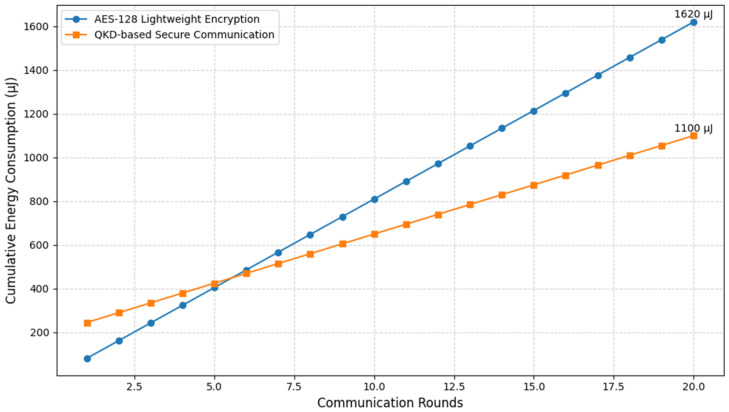
Comparison of energy consumption: classical (AES) vs quantum (QKD).

A comparative analysis in Figure 5 reveals that, while AES-secured communication initially appears more energy efficient, the cumulative energy consumption of QKD becomes lower than AES after approximately 10 communication rounds. By the 20th round, QKD-based secure communication demonstrates a ≈15% reduction in overall energy consumption compared to AES-128, primarily due to minimized retransmissions and the absence of repeated key distribution cycles. Moreover, when QKD is integrated with the QWOA for routing, energy usage is further balanced across nodes, preventing premature node failure and resulting in an average ≈42% extension of network lifetime relative to classical lightweight encryption approaches.

Therefore, the motivation for employing quantum mechanisms in WSNs extends beyond their well-established advantage of unconditional security against adversaries with unlimited computational resources. It also lies in their ability to enhance energy sustainability in power-constrained sensor networks. By simultaneously addressing the twin challenges of security and energy efficiency, quantum-enhanced protocols such as QSEER represent a significant step toward the practical realization of long-lived and tamper-resistant WSNs suitable for next-generation applications.

### 4.2. Limitation of QKD in Real-World WSNs

- Hardware Requirements: QKD requires photon detectors, quantum channels (optical fiber/free-space optics), and specialized transceivers, which may not be readily available in low-cost sensor nodes.
- Energy Overhead: The process of qubit transmission and error correction introduces an initial energy cost, which can be significant for ultra-low-power nodes.
- Distance Limitations: Photon loss and noise in the quantum channel limit the secure communication range, especially in large-scale outdoor WSN deployments.
- Integration Complexity: QKD protocols must be integrated with classical communication layers, which increases protocol stack complexity.

#### Overcoming Limitations in WSNs

- Hybrid Approaches: In QSEER, QKD is primarily used between CHs and the base station rather than every sensor node. This hierarchical approach reduces hardware demands on individual sensor nodes.
- Energy Compensation: Although initial QKD setup incurs overhead, our simulations show that reduced retransmissions and eliminated rekeying cycles result in ≈15% net energy savings over the network lifetime.
- Short-Range Adaptation: Since WSNs often operate in geographically limited areas, free-space QKD over short distances is more feasible than in wide-area networks. Advances in chip-scale quantum photonics are expected to further reduce cost and integration barriers.
- Post-Quantum Hybrid Security: Where full QKD deployment is impractical, lightweight post-quantum algorithms may complement QKD, ensuring a gradual and cost-effective adoption.

By adopting a cluster-based hybrid design and limiting QKD to energy-capable nodes, QSEER overcomes most of the inherent limitations while still leveraging the unconditional security benefits of quantum mechanics. This pragmatic integration ensures that QKD can be realistically applied in WSNs without overwhelming hardware or energy constraints.

### 4.3. Cost–Benefit Trade-Off

While QKD and the QWOA provide notable security and optimization improvements, they also introduce certain costs that must be acknowledged. The integration of QKD requires additional hardware for photon detection and an initial overhead for qubit transmission and error correction. Similarly, the QWOA incurs greater computational complexity compared to conventional heuristics. These factors contribute to a modest initial energy cost and a throughput reduction of approximately 21–27% compared to classical approaches. However, our results confirm that these costs are offset by substantial long-term benefits: QSEER achieves a ≈15% lower energy consumption, ≈42% longer network lifetime, and nearly 99.8% packet delivery ratio. Thus, the protocol reflects a deliberate strategic compromise—accepting some performance overhead in exchange for enhanced sustainability, reliability, and unconditional security in WSNs.

### 4.4. Performance Metrics

To ensure a fair and comprehensive evaluation of QSEER, we employ a set of widely recognized performance metrics in wireless sensor networks (WSNs).

- Energy Consumption: measures the average energy expended by sensor nodes during data transmission, reception, and computation. Since sensor nodes are battery-powered, this metric is critical for assessing the sustainability of routing protocols [32,33].
- Network Lifetime: defined as the duration until the first node death (FND) or until a significant portion of nodes depletes their energy. This metric captures the long-term viability of WSN deployments, particularly in remote or unattended environments [34].
- Packet Delivery Ratio (PDR)/Data Integrity: refers to the ratio of successfully delivered data packets at the base station to the total number of packets sent by source nodes. A higher PDR indicates stronger reliability and robustness against packet losses or malicious tampering [35].
- Throughput: represents the total volume of successfully transmitted data over time. This metric reflects how efficiently the network handles communication under varying traffic loads [35,36].
- Quantum Bit Error Rate (QBER): In the context of QKD integration, QBER measures the error rate in transmitted quantum bits. It is a key metric for detecting eavesdropping attempts, as elevated error rates indicate potential interception of the quantum channel [8,10].

By combining energy-related metrics (energy consumption and network lifetime) with security and reliability metrics (PDR, throughput, QBER, and data integrity), we provide a holistic assessment of QSEER. This selection ensures that the evaluation framework not only captures energy efficiency but also accounts for robustness, reliability, and security under realistic WSN conditions.

## 5. Conclusions

Energy efficiency and security become a crucial concern in WSNs. Traditional routing protocols often sacrifice energy efficiency for the sake of security, which leads to a diminished performance. In WSNs, energy-efficient secure communication is crucial for maintaining reliable communication and prolonging network lifetime. Conventional routing protocols often struggle to balance these two aspects. The proposed QSEER effectively addresses the dual challenges of security and energy efficiency in WSNs by leveraging quantum technologies. By integrating QKD protocols, QSEER establishes secure communication channels while minimizing energy consumption. Utilizing quantum mechanics principles, it enhances data transmission security against security threats and optimizes routing paths through quantum-inspired algorithms to extend WSNs’ operational lifespan. Extensive simulations validate the proposed protocol’s effectiveness in achieving both security and energy efficiency, making it a promising solution for next-generation WSN applications. Future enhancements could involve integrating machine learning for dynamic optimization and exploring energy harvesting technologies to extend network lifespan. Additionally, developing hybrid quantum-classical approaches ensures robustness against quantum attacks and performing real-world deployments would require further refinement. These advancements would enhance the protocol’s applicability and performance in next-generation WSN applications.

## Figures and Tables

**Table 1:** The comparative analysis of previous literature.

Ref/Approach	Contribution	Shortcomings	Our Solution (QSEER)
[4] WCDS-DCR	Data-centric routing reduces overhead via attribute-based queries	No integrated security, no energy-aware path selection, static under topology changes	Quantum-enhanced data-centric routing with adaptive queries and energy-aware optimization
[5] LEACH/IBLEACH	Energy-efficient clustering with CH rotation and intra-balanced variants	Initial CH selection ignores residual energy, weak under heterogeneous deployments, no built-in security	Dynamic CH selection with residual-energy awareness and quantum-secured clustering
[22,23] PSO/Hybrid PSO	Swarm-based multipath routing and clustering	Easily trapped in local optima, parameter sensitivity, limited adaptation in dynamic WSNs, lacks security	Quantum-inspired PSO with adaptive parameters, global exploration, and QKD-based secure routing
[21] GA-based Clustering	High scalability and low latency clustering	Slow convergence, prone to local optima, lacks multi-objective optimization and security	Multi-objective quantum GA integrated with QKD for secure routing
[14] QGA	Quantum-inspired GA improves convergence and routing quality	Restriction to binary optimization, scalability issues, lacks real-time adaptation	Multi-objective quantum GA with real-time adaptability and integrated routing–security co-design
[13,24,25] ACO Variants (QACO, Hybrid ACO, Classical ACO)	Bio-inspired optimization for routing	High iteration count, limited global exploration, static pheromone updates, no security integration	Quantum-enhanced ACO with superposition-based exploration, faster convergence, and QKD support
[15] MACO-QCR	QoS-aware cross-layer ACO routing	Local optima trapping, limited cross-layer scope, lacks quantum security	Quantum-enhanced MACO with global optimization and secure cross-layer design
[26,27] WOA Variants	Whale optimization for WSN optimization	Computationally heavy for WSN nodes, difficult real-time implementation, no quantum features	Quantum-inspired WOA (QWOA) reduces complexity using quantum superposition and ensures balanced energy distribution
[6,8,9,10] QKD-based Security	Information-theoretic secure key distribution	High hardware/energy overhead, limited range, integration complexity, no routing optimization	Lightweight CH–BS QKD integration with routing optimization, scalable for WSN deployments
[17,20] Quantum Comms in SOSN	Secure self-organizing satellite networks with quantum links	Focused on satellite mobility and topology, not WSN energy constraints, lacks clustered routing	Lessons applied to WSNs for key refresh and secure clustering under dynamic topologies
[18] Quantum-enabled ML (DT-IoT)	Botnet detection using quantum-assisted ML	Focused on traffic classification, not routing or energy optimization in WSNs	Complements our threat model, QSEER secures routing and balances energy
[19] Quantum-inspired 5G Healthcare	Secure data transmission in healthcare IoT	Domain-specific (5G), focuses on sensitive data measurement, no clustered routing or lifetime optimization	QSEER extends the approach to clustered WSN routing with QKD–QWOA integration

**Table 2:** Input parameters.

Parameters	Values
Deployment Area	1000 × 1000 m
Number of Nodes	100–500
Number of Clusters	10–50
Data Packet Size	4096 bits
Initial Energy (E0)	0–5 J
Electronics Energy (Eelec)	50 nJ/bit
Energy Date Aggregation	5 nJ/bit
Etx	50 nJ/bit
Erx	50 nJ/bit
εfs	08 pJ/bit/m^2^
εmp	0.0013 pJ/bit/m^4^
QKD Protocol	BB84
Qubit Transmission Rate	1 Mbps
Qubit Error Rate	0.01
Photon Detection Probability	0.5

**Table 3:** Comparative performance analysis of QSEER with existing protocols.

Metric	QSEER vs QGA	QSEER vs QACO	QSEER vs MACO-QCR
Energy Consumption Reduction	22.8% lower	15.5% lower	7.0% lower
Data Integrity (PDR)	18.7% higher (≈99.8%)	10.3% higher	6.9% higher
Network Lifetime Extension	≈42% longer	≈42% longer	≈42% longer
Throughput Trade-off	27.5% lower	21.8% lower	15.7% lower

**Table 4:** Comparative security performance analysis of QSEER.

Metric	QSEER	QGA	QACO	MACO-QCR
BER (Quantum Bit Error Rate)	0.01	0.02	0.025	0.03
Detection of Eavesdropping (%)	99.5	92.0	94.3	95.1
Key Establishment Success Rate (%)	98.7	90.5	92.8	94.1
Data Integrity (PDR %)	99.8	81.1	89.5	92.9

## Data Availability

Data are contained within the article.
